# Anatomical characteristics and potential gene mutation sites of a familial recurrent patellar dislocation

**DOI:** 10.1186/s12920-022-01330-9

**Published:** 2022-08-07

**Authors:** Qi-hao Zhang, Yan Zhang, Rui-xuan He, Han-ming Guo, Xin-guang Wang

**Affiliations:** Department of Orthopedics, Huizhou Central People’s Hospital, Huizhou, 516008 Guangdong People’s Republic of China

**Keywords:** Patellar dislocation, Exome sequencing, Sanger sequencing, Pathogenic gene

## Abstract

**Background:**

Recurrent patellar dislocation is the result of anatomical alignment and imbalance of restraint of bone and soft tissue. We investigate the anatomical characteristics of the knee joint in a family of patients with recurrent patella dislocation, and to screen the possible pathogenic genes in this family by whole exome sequencing in 4 patients and 4 healthy subjects, so as to provide theoretical basis for the pathogenesis of this disease.

**Methods:**

The data related to patella dislocation were measured by imaging data. The peripheral blood DNA of related family members was extracted for the whole exome sequencing, and then the sequencing results were compared with the human database. By filtering out synonymous variants and high-frequency variants in population databases, and then integrating single nucleotide non-synonymous variants of family members, disease-causing genes were found.

**Results:**

All patients in this family have different degrees of abnormal knee anatomy, which is closely related to patella dislocation. The sequencing results of patients and normal persons in this patella dislocation family were compared and analyzed, and the data were filtered through multiple biological databases. Find HOXB9 (NM_024017.4:c.404A>G:p.Glu135Gly),COL1A1(NM_000088.3:c.3766G>A:p.Ala1256Thr),GNPAT(NM_014236.3:c1556A>G:p.Asp519Gly),NANS(NM_018946.3:c.204G>C:p.Glu68Asp),SLC26A2(NM_000112.3:c.2065A>T:p.Thr689Ser) are nonsynonymous variants (MISSENSE). Through Sanger sequencing, the identified mutations in HOXB9 and SLC26A2 genes were only present in samples from patients with recurrent patellar dislocation.

**Conclusions:**

The patients with recurrent patellar dislocation had markedly abnormal knee anatomy in this family. HOXB9 gene and SLC26A2 gene were found to be the possible pathogenic genes or related genes for patella dislocation.

## Background

Recurrent patellar dislocations, also known as habitual patellar dislocation, are the result of anatomical alignment and imbalance of restraint of bone and soft tissue [1]. Recurrent patella dislocation is a common knee joint injury in adolescents with immature bones. After the first attack, the incidence of ipsilateral recurrence was 36% and contralateral dislocation was 5% [2]. The clinical manifestations are knee pain, swelling and repeated lateral dislocation of the patella [3]. The injury caused by long-term and repeated dislocation of patella in patients with recurrent patellar dislocation will cause chronic degenerative changes of knee joint, and the psychological impact caused by repeated dislocation of patella will seriously affect the quality of life of patients’ lives. The etiology of recurrent patellar dislocation is complicated, most of which are related to abnormal anatomical structure or dysplasia of knee joint [4]. Some studies have suggested that recurrent patellar dislocation has certain genetic predisposition, but there are no studies on the pedigree of recurrent patellar dislocation, gene loci and downstream gene expression mechanism at home and abroad.

With the development of high-throughput sequencing technology, whole-exome sequencing is increasingly used in the research of Mendelian diseases and miscellaneous diseases. Whole exome sequencing refers to a genome analysis method which uses sequence capture technology to capture and enrich the DNA in the whole genome exome region, and then perform high-throughput sequencing. Although the human exome region only accounts for 1% of the entire genome sequence, about 85% of disease-causing variants are located in this region [5]. Whole exome sequencing has the following advantages: (1) The cost of whole exome sequencing is relatively low. (2) Since there are few sequences to be sequenced, the sequencing time is shorter, which improves the research efficiency. (3) Exome sequencing, regardless of the size of the sample, whether it is from the same family or not, the sequencing integration analysis can be performed. (4) Exome sequencing can more accurately determine candidate genes more accurately, which provides convenience for subsequent screening and identification of gene functions. Exome sequencing has broad prospects in the genetic diagnosis of diseases and the research of disease-causing genes, and has been widely used in Mendelian diseases, cancers and complex diseases. Tsz Kin Ng et al. revealed 8 novel USH2A variants in Chinese patients with familial and sporadic retinitis pigmentosa through whole exome sequencing technology, which is helpful for clinical diagnosis of Usher syndrome 2 from patients with sporadic retinitis pigmentosa [6]. Marjan SHAKIBA and Mohammad KERAMATIPOUR performed a systematic search on well-known databases such as Google, Medline, PubMed, Cochrane, etc. From the search data, it can be seen that Whole Exome Sequencing is an effective and useful technology for diagnosing metabolic and neurogenetic diseases, especially in complex or unresolved cases [7]. The use of Exome sequencing to find disease-related disease-causing genes and variant sites, and explore their pathogenic mechanisms, is of far-reaching significance for the early diagnosis of the disease and early therapeutic intervention.

In this study, we collected information, imaging data and blood samples of a family member of a recurrent patellar dislocation, to understand the anatomical characteristics of the knee joint of the affected member of the family, and draw a family tree to analyze the possible genetic characteristics of the family's patellar dislocation. We then use whole-exome sequencing to find possible pathogenic genes for patellar dislocation, and provide a theoretical basis for the pathogenesis of the disease.

## Methods

### Patients

The subjects of this study were 52 people from the same family, including 13 patients. Through the family member's medical history, physical examination and imaging examination, two deputy chief physicians or chief physicians made a diagnosis of the disease for family members with recurrent patellar dislocation.

### Anatomy measurement

**Fig. 1:**
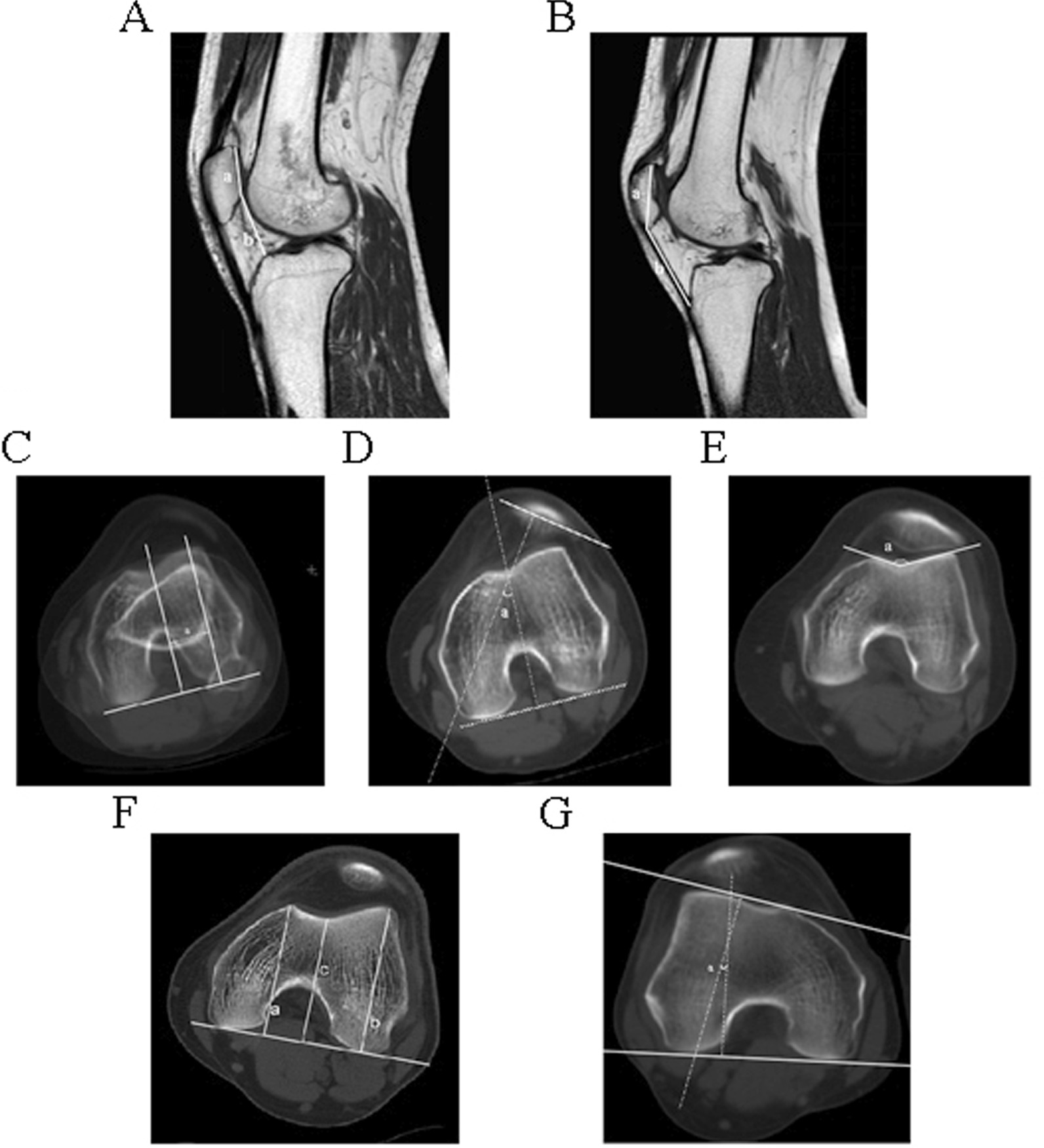
The anatomical data measurement of the patient's knee joint. **A** Insall-Salvati index. **B** Caton-Deschamps index. **C** Tibial tubercle–trochlear groove. **D** Patellar Tilt. **E** Trochlear sulcus angle. **F** Trochlear sulcus depth. **G** Trochlear angle

We collect the patient's imaging data and measure related anatomical values to understand the anatomical structure of the knee joint and its relationship with the dislocation of the patella. Figure 1 shows anatomic measurements of the patient's knee joint. The relevant values measured include the trochlear dysplasia classification (Dejour) [8], the trochlear angle [9, 10], the trochlear sulcus angle, the trochlear sulcus depth [11], the insall-Salvati index [12], the caton-Deschamps index [13], the tibial tubercle–trochlear groove (TT–TG) distance [14], the patellar Tilt [15] and the Q angle.

### DNA extraction, library construction and whole exome sequencing

There are 8 samples in this study, including 4 patients and 4 healthy people from the family. Among them, numbers 1–4 are patients, and 5–8 are healthy people. After obtaining their signed consent, 3 ml of their peripheral blood was taken and sent to Wuhan Huada Medical Laboratory Co., Ltd. for DNA extraction and exome sequencing. The genomic DNA of 4 patients and 4 healthy people was prepared as an BGISEQ sequencing library, and the sequencing libraries were then enriched for the desired target using the BGISEQ Exome Enrichment protocol. The captured libraries were sequenced using an BGISEQ-500 Sequencer.

### Bioinformatic analysis

The raw sequencing data was processed using the following steps (1) Removing reads.

containing sequencing adapter; (2) Removing reads whose low-quality base ratio (base quality less than or equal to 5) is more than 50%; (3) Removing reads whose unknown base ('N' base) ratio is more than 10%. First, the clean data was produced by data filtering on raw data. All clean data of each sample was mapped to the human reference genome (GRCh37/hg19). Burrows-Wheeler Aligner (BWA) software was used to do the alignment. Local realignment around InDels and base quality score recalibration were performed using GATK, with duplicate reads removed by Picard tools. The sequencing depth and coverage for each individual were calculated based on the alignments. All genomic variations, including SNPs and InDels were detected by the HaplotypeCaller of GATK(v3.3.0). After that, the hard-filtering method was applied to get high-confident variant calls. Then the SnpEff tool (http://snpeff.sourceforge.net/SnpEff_manual.html) was applied to perform a series of annotations for variants. Finally, the data of annotation results were analyzed, and the suspected pathogenic variants were screened using CLINVAR, OMIM and HGMD databases.

### Screening of pathogenic mutant genes

Synonymous variants were removed based on variant annotation results, and non-synonymous variants, splicing variants and frameshift variants were concerned. Then, by comparison, the high-frequency variants with MAF ≥ 1% in the Thousand Genome Database and EXAC Database were removed. The harmful variants were predicted by means of SIFT, PolyPhen2, variant assessor, etc., and the variants that were present in family patients but not in normal controls were preserved.

### Single nucleotide variation was detected by Sanger sequencing

Specific primers were designed according to the variant sites of candidate genes. PCR amplification kit (Takara Company) was used to amplify the target sequence, and Sanger sequencing was performed by Wuhan Huada Medical Laboratory Co., Ltd., and the sequencing results were compared with the reference sequence in the GenBank database.

## Results

### Family data and genealogy analysis

**Fig. 2:**
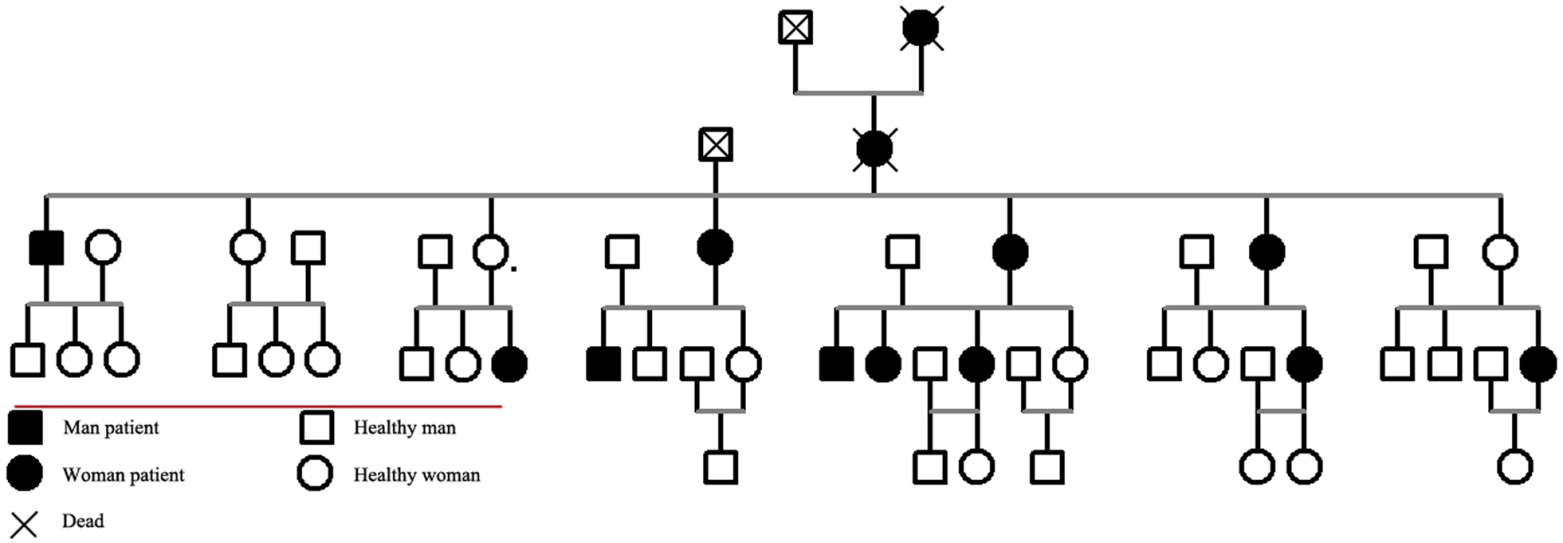
A family pedigree of recurrent patella dislocation

There are 52 people in the family, including 13 patients, 4 of whom have died. There is no history of obvious trauma. According to the family investigation and diagnosis, the genealogy was drawn, as shown in Fig. 2. Genealogical analysis shows that there are five generations of the family, with cases occurring in each generation, and both men and women may be affected in each generation. As the fifth generation members are all children, no patients have been found. The family accords with the characteristics of autosomal recessive inheritance.

### Anatomical characteristics of the patella femur in the family

According to the wishes of members of the family, we collected data on 14 knees in 8 patients. Through imaging analysis and measurement, we studied the anatomical features of the knee joint of this family patient. From trochlear dysplasia classification (Dejour), we found 2 cases of type A, 5 cases of type B, 6 cases of type C, and 3 cases of type D. It can be seen from Table 1 that the patient's trochlear angle is 16.0 ± 3.9°, the trochlear sulcus angle is 165.8 ± 8.7°, and the trochlear sulcus depth is 1.54 ± 1.25 mm. These measurement data fully prove that the patient has obvious pulley dysplasia. In addition, related studies have found that the pulley angle is significantly positively correlated with Type B to Type D of Dejour classification. Patella alta is a risk factor for dislocation of patella. Insall-Salvati index and Caton-Deschamps index are commonly used indicators for the diagnosis of patella alta. By measurement, Insall-Salvati index and Caton-Deschamps index values were both 1.3 ± 0.1 mm, which were consistent with the diagnosis of patella alta. Patella tilt is one of the risk factors affecting knee joint stability. The abnormal trajectory of the patella can be measured by the tilt of the patella. Our study found that the tilt angle of the patient's patella is 25.3 ± 6.2°, which is significantly larger than the normal value. Important factors related to patella stability are the location of the tibial tubercle and Q Angle. The tibial tubercle–trochlear groove (TT–TG) distance is a common method to evaluate the position of the tibial nodule. In this family, the TT–TG value was 25 ± 4 mm and the Q Angle was 20.4 ± 2.3°, which was also significantly higher than the normal value. (Table 1).

**Table 1:** Anatomical data of the patella femur in the family

Samples	Side	Trochlear angle (°)	Trochlear sulcus angle (°)	Trochlear sulcus depth (mm)	ISI	CDI	TT–TG (mm)	Patellar Tilt (°)	Q angle (°)	TDC
1	Left	19.2	178.7	0.1	1.3	1.3	27.2	28.2	22.2	D
	Right	18.6	171.8	0.1	1.4	1.5	24.3	27.3	21.7	D
2	Left	12.6	158.3	3.0	1.3	1.6	28.1	21.0	20.4	B
	Right	15.5	162.3	2.5	1.3	1.5	27.3	24.7	21.2	C
3	Left	9.2	152.5	3.2	1.0	1.1	18.5	18.2	15.1	A
	Right	11.3	159.7	2.6	1.1	1.1	28.2	23	19.4	B
4	Right	18.9	169.3	2.4	1.3	1.3	28.3	24.3	22.8	C
5	Left	23.2	179.0	0.1	1.2	1.3	30.1	26.7	20.2	D
	Right	17.7	171.1	0.2	1.4	1.4	25.6	24.8	19.3	C
6	Left	18.4	172.1	0.2	1.5	1.3	26.2	45.6	24.2	C
	Right	16.4	168.8	0.1	1.5	1.4	24.8	23.6	23.3	C
7	Left	10.1	153.2	2.9	1.2	1.2	17.8	21.6	18.1	A
	Right									
8	Left	13.6	154.2	2.4	1.2	1.4	25.2	21.6	18.2	B
	Right	18.6	169.5	1.3	1.4	1.4	25.9	23.6	19.3	C
Mean + SD	16.0 ± 3.9	165.8 ± 8.7	1.5 ± 1.3	1.3 ± 0.1	1.3 ± 0.1	25.5 ± 3.4	25.3 ± 6.2	20.4 ± 2.3	/	

*ISI* Insall–Salvati index, *CDI* Caton–Deschamps index, TT–TG Tibial tubercle–trochlear groove, *TDC* Trochlear dysplasia classification (Dejour)

### Data production

To discover genetic variations in this project, we performed whole exome sequencing of 8 DNA sample(s). After removing low-quality reads we obtained on average 469,919,789 clean reads (23,495.99 Mb). The clean reads of each sample had high Q20 and Q30, which showed high sequencing quality. The average GC content was 45.27%. The average sequencing depth of the target region was about 133.58X. All whole exome sequencing data production was summarized in Table 2.

**Table 2:** Statistical table of exon sequencing data

Samples	Clean reads	Clean bases (Mb)	Q20 (%)	Q30 (%)	GC content (%)
1	524,407,906	26,220.40	98.00	91.09	45.61
2	525,486,666	26,274.33	97.92	91.37	43.25
3	398,169,744	19,908.49	97.86	91.32	44.56
4	487,379,870	24,368.99	97.69	90.62	44.48
5	516,845,386	25,842.27	97.77	91.11	45.71
6	426,657,740	21,332.89	97.27	89.80	45.73
7	461,942,166	23,097.11	97.62	90.75	45.33
8	418,468,840	20,923.44	97.46	90.15	47.52
Average	469,919,789	23,495.99	23,155.44	90.98	45.27

Clean reads—total reads after filtration; clean bases—total data amount after filtering low-quality reads from sequencing data; Q20—proportion of bases with a mass value greater than 20; Q30—proportion of bases with a mass value greater than 30; GC content—ratio of sequencing data GC

### Screening for pathogenic genes and validation using sanger sequencing.

**Fig. 3:**
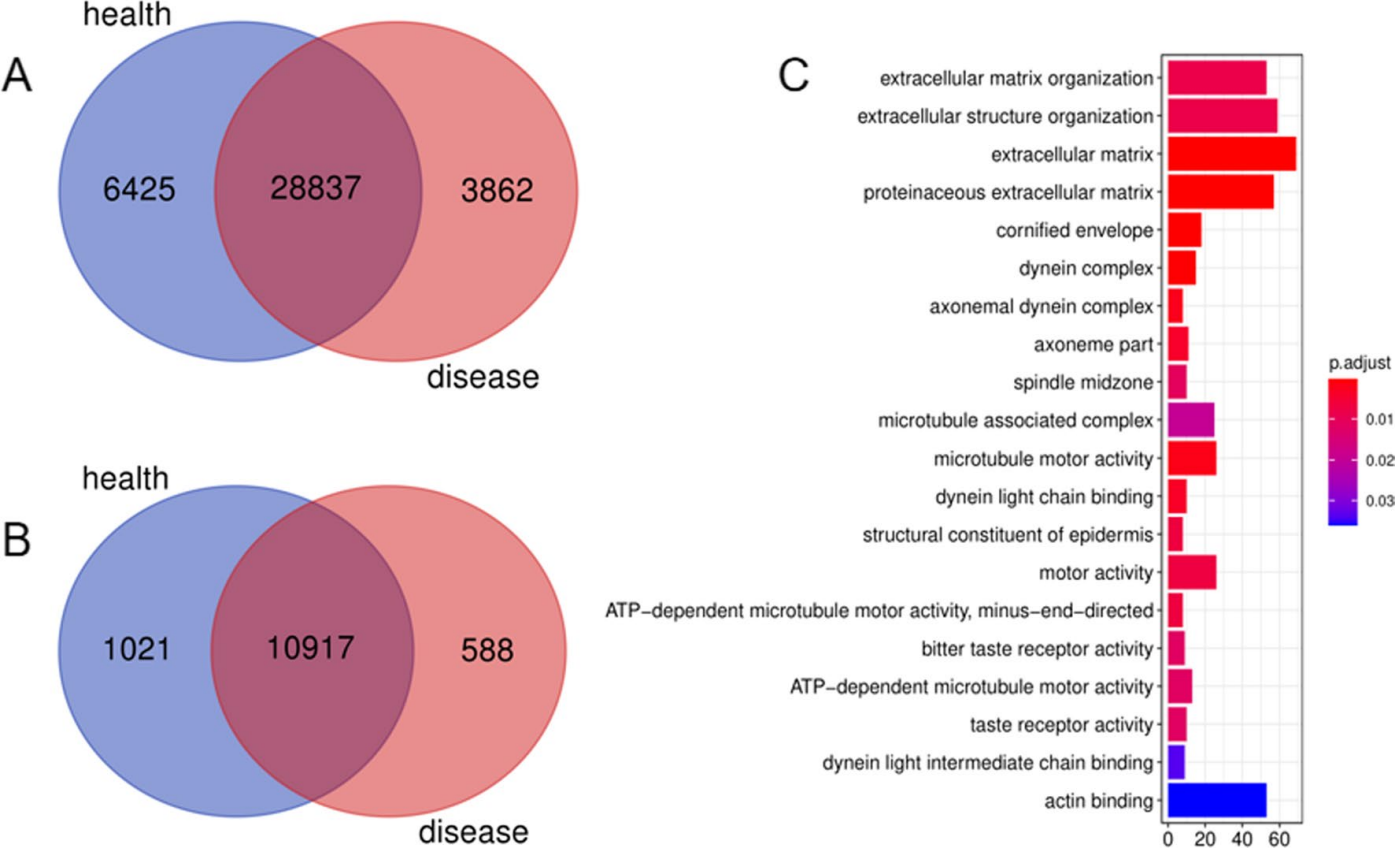
Screening and functional enrichment analysis of mutant genes. **A** Venn diagrams of common and unique variant information for both disease and health groups; **B** Venn diagram of the gene on the annotation of the variant site; **C** gene GO function enrichment map of disease group-specific variant site annotation

**Fig. 4:**
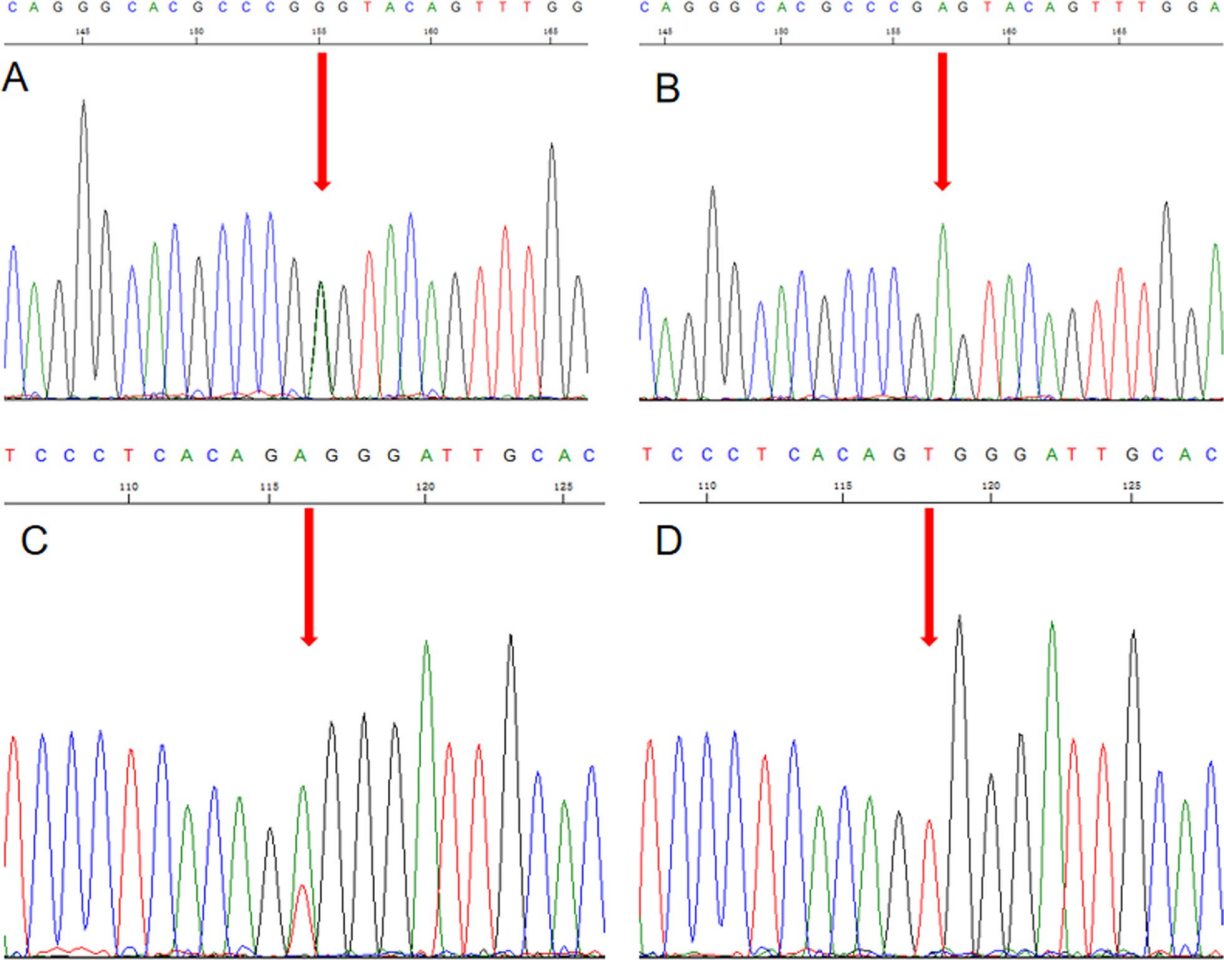
Sanger sequencing results of HOXB9 and SLC26A2 genes. **A** sequencing peak figure of HOXB9 gene c.404A>G heterozygous variant in patients; **B** sequencing peak figure of the normal HOXB9 genotype in healthy samples; **C** sequencing peak figure of c.2065A>T heterozygous variant in SLC26A2 gene in patients; **D** sequencing peaks figure of SLC26A2 genotypes in healthy samples

Exome sequencing samples included 4 patients and 4 healthy subjects in this family of recurrent patella dislocation. 3862 unique variants were identified in the disease group (Fig. 3A), these variants were annotated to 588 genes by annovar (Fig. 3B). Gene Ontology and Kyoto Encyclopedia of Genes and Genomes Analysis of the 588 Genes show that most of these genes enrichment to extracellular matrix organization, cornified envelope, dynein complex, axonemal dynein complex and joint related pathways (Fig. 3C). At the same time, we searched for multiple epiphyseal dysplasia and chondrodysplasia signaling pathway-related genes in the OMIM and HGMD databases, and finally we screened out 5 candidate mutant genes, namely: HOXB9(NM_024017.4:c.404A>G:p.Glu135Gly),COL1A1(NM_000088.3:c.3766G>A:p.Ala1256Thr),NPAT(NM_014236.3:c.1556A>G:p.Asp519Gly),NANS(NM_018946.3:c.204G>C:p.Glu68Asp),LC26A2(NM_000112.3:c.2065A>T:p.Thr689Ser).Validation by sanger first-generation sequencing revealed that the HOXB9 gene could detect the c.404A>G variant in 3 patient samples, and the SLC26A2 gene could detect the c.2065A>T variant in 1 patient (Fig. 4). Therefore, we believe that mutations in HOXB9 and SLC26A2 genes may be associated with recurrent patellar dislocation in this family.

## Discussion

The instability of the patellofemoral joint is closely related to the abnormal anatomy of the knee joint, including trochlear dysplasia, high patella, and balance of medial and lateral retinaculum. Under normal conditions, the knee remains stable. In the absence of violence, the patella does not dislocate. In this study, patellar dislocation occurred in all patients without injury. We collected comprehensive data on these patients, including weight, height, age and the time of the first patellar dislocation, etc., but the impact on this study was not significant, so we did not analyze too much. We focused on the site of the disease, conducted a comprehensive imaging examination of the knee joint and collected relevant anatomical data. According to imaging data and anatomical data (Fig. 1), abnormal anatomical structure of knee joint is the main cause of patellar dislocation.

There are few familial reports of patellar dislocation, and there are few studies on the pathogenic genes that may exist in its onset. CM Jimmy Chan et al. reported a familial recurrent patellar dislocation in 2018, which was considered autosomal dominant. Genotypic assessment suggested that the family pathogenesis was related to the balanced translocation of chromosomes 15 and 20 [16]. In this study, from the first generation to the third generation (Fig. 2), we can see that the family is autosomal recessive, but X-linked dominant inheritance cannot be ruled out. However, there are female patients in the fourth generation, but none of their parents have the disease. So we can rule out X-linked dominant inheritance. Therefore, we believe that the family of patellar dislocation is autosomal recessive. Then through whole exome sequencing and Sanger verification, we found that the identified mutations in HOXB9 and SLC26A2 genes were only present in samples from patients with recurrent patellar dislocation. This indicates that it may be a related pathogenic gene.

SLC26A2 is essential for chondrocyte proliferation and differentiation as well as proteoglycan synthesis, and can regulate the final stage of chondrocyte size expansion. Park M et al. found that SLC26A2-mediated protein sulfuration plays an important role in cellular signaling, which is associated with abnormal cartilage development [17]. The allele mutation of SLC26A2 often causes diastrophic dysplasia, so it is also called diastrophic transporter gene. This gene is located on distal chromosome 5q and encodes a sulfate transporter. This protein absorbs sulfate into chondrocytes and plays an important role in endochondral bone formation [18].

Homeobox (HOX) genes are a group of 39 related genes that encode conserved transcription factors related to vertebrate bone development. The HOXD9 gene not only regulates the growth and differentiation of muscle cells, but also the differentiation of mesenchymal cells into new bone and cartilage. Studies have found that it has a great correlation with acetabular shape, the ossification groove development and the position of the femoral head [19].

We obtained several disease-related candidate genes such as HOXB9, COL1A1, GNPAT, NANS, and SLC26A2 by whole exome sequencing analysis of the disease group and healthy group samples. Then, through Sanger sequencing, it was found that HOXB9 and SLC26A2 were only mutated in the disease group. In addition, the functions of these two genes were related to bone growth and development, so it was inferred that they may be pathogenic genes or related genes in this family. Although SLC26A2 and HOXD9 variants may lead to systemic skeletal abnormalities, no other related structural abnormalities outside the knee joint were found in this family. We consider patellar dislocation to be a multifactorial cause. In addition to skeletal changes, various factors such as the imbalance of the medial and lateral retinaculum and related soft tissues, the effect of gravity, and large activities make the patient only show symptoms of knee dislocation. Or the pathogenic gene is more expressed in patellar dislocation. Of course, in our current research sample, due to various factors, specimens from all family members have not been collected, and the existence of sporadic cases cannot be ruled out, which requires us to continue to follow up and further verify.

## Conclusions

Overall, this study found multiple patients with recurrent patellar dislocation in a family. The disease is autosomal recessive in this family, and most patients have structural abnormalities of varying degrees in the knee joints. Through exome sequencing and gene screening, HOXB9 gene and SLC26A2 gene were found to be the possible pathogenic genes or related genes for patella dislocation, which provided a basis for our in-depth study of the disease and future therapeutic targets.

## Data Availability

The datasets generated for this study can be found in the NCBI SRA accession PRJNA766026.
